# First person – Helen Molteni

**DOI:** 10.1242/dmm.052832

**Published:** 2026-02-20

## Abstract

First Person is a series of interviews with the first authors of a selection of papers published in Disease Models & Mechanisms, helping researchers promote themselves alongside their papers. Helen Molteni is first author on ‘A small-molecule screen identifies that elafibranor links mechanical cues and Irf6-dependent epithelial differentiation’, published in DMM. Helen is a Craniofacial Surgeon-Scientist Training Fellow in the lab of Eric Liao at Children's Hospital of Philadelphia, Philadelphia, PA, USA, investigating genetic drivers of orofacial clefts, emphasizing the role of epithelial differentiation genes in midface development.


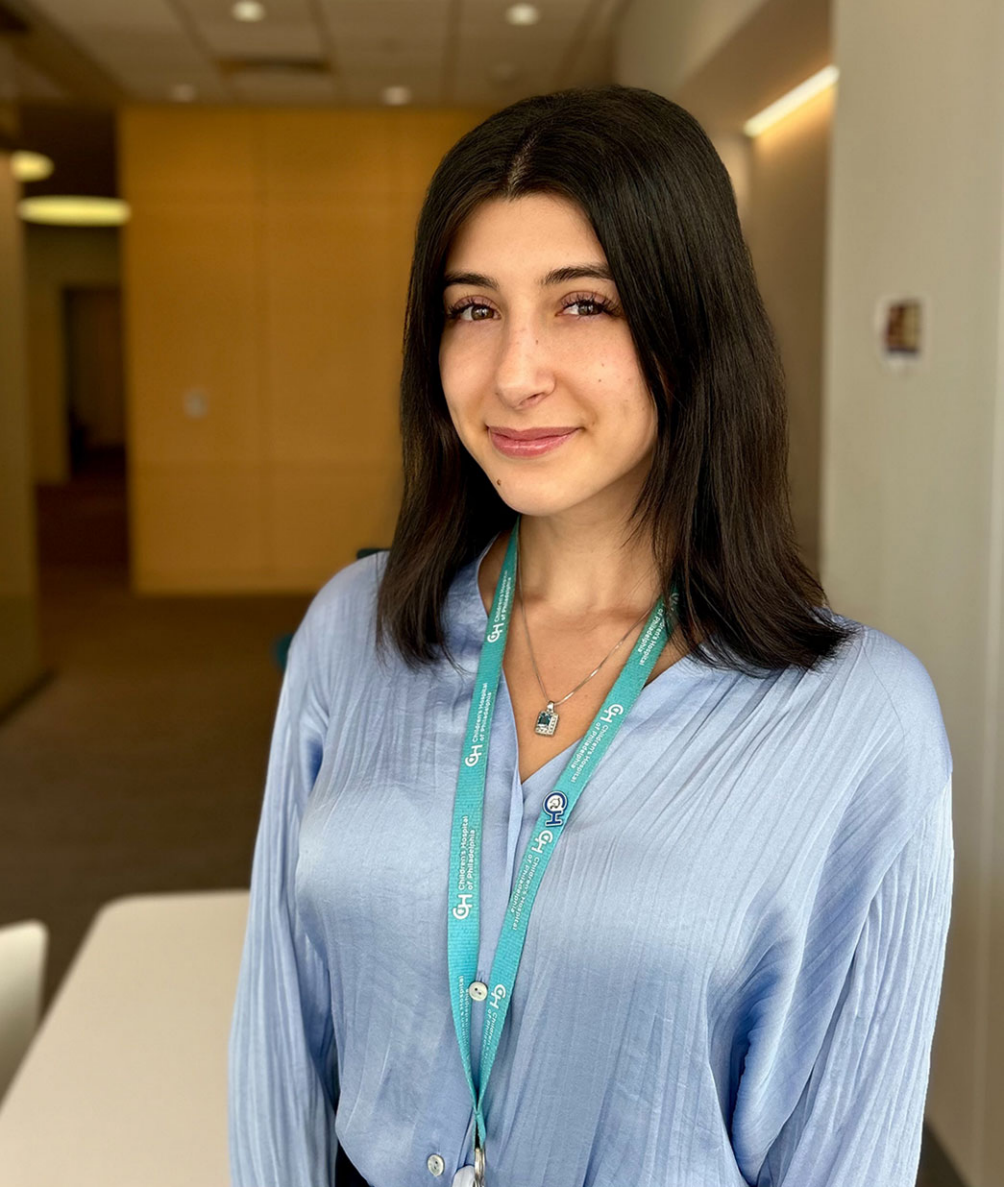


**Helen Molteni**

**Who or what inspired you to become a scientist?**

My initial interest in science and medicine was inspired by my grandmother, Dr Loredana Brizio-Molteni, who was one of the first women surgeon-scientists in the USA. When I was little, she would always get me science-related gifts for Christmas. Although she passed while I was still in elementary school, during college, my grandfather would send me copies of papers and textbooks she authored.

My grandmother dedicated her life to bridging the gap between the lab bench and the operating room, employing fundamental discoveries to inform and improve patient care. I am proud to honour her legacy through my own translational research efforts while beginning my career as an aspiring surgeon-scientist.

**What is the main question or challenge in disease biology you are addressing in this paper? How did you go about investigating your question or challenge?**

Our work seeks to better understand the *IRF6* gene regulatory network as it pertains to epithelial differentiation and craniofacial development. Specifically, we were interested in identifying potential modifiers of IRF6-dependent epithelial differentiation. To accomplish this aim, we performed a forward genetic screen of nearly 600 different small molecules using an *irf6* maternal-null zebrafish model. Embryos lacking maternal Irf6 possess a fragile, hyperpermeable epithelial barrier, resulting in lethality prior to gastrulation. We therefore sought to identify small molecules that could delay lethality in this model by restoring epithelial integrity. Through the chemical screen and follow-up phenotypic analyses, we found that elafibranor, a dual PPARα/PPARβ agonist, promotes IRF6-dependent epithelial integrity by restoring localization of tight junctions.

**How would you explain the main findings of your paper to non-scientific family and friends?**

*IRF6* is one of the major genes responsible for orofacial clefts, and it is involved in development of the skin. When this gene is mutated, the early skin barrier becomes weak and fragile. In our study, we found that a drug called elafibranor can strengthen the early skin barrier by helping secure attachments between skin cells. However, in healthy, non-mutated developing skin, this strengthening may come at the expense of reduced skin plasticity and actually constrict development of the underlying facial structures.

[Our] findings provide new insights into the pathogenesis of skin barrier defects and epithelial anomalies present in syndromic orofacial clefts attributable to *IRF6*

**Figure:**
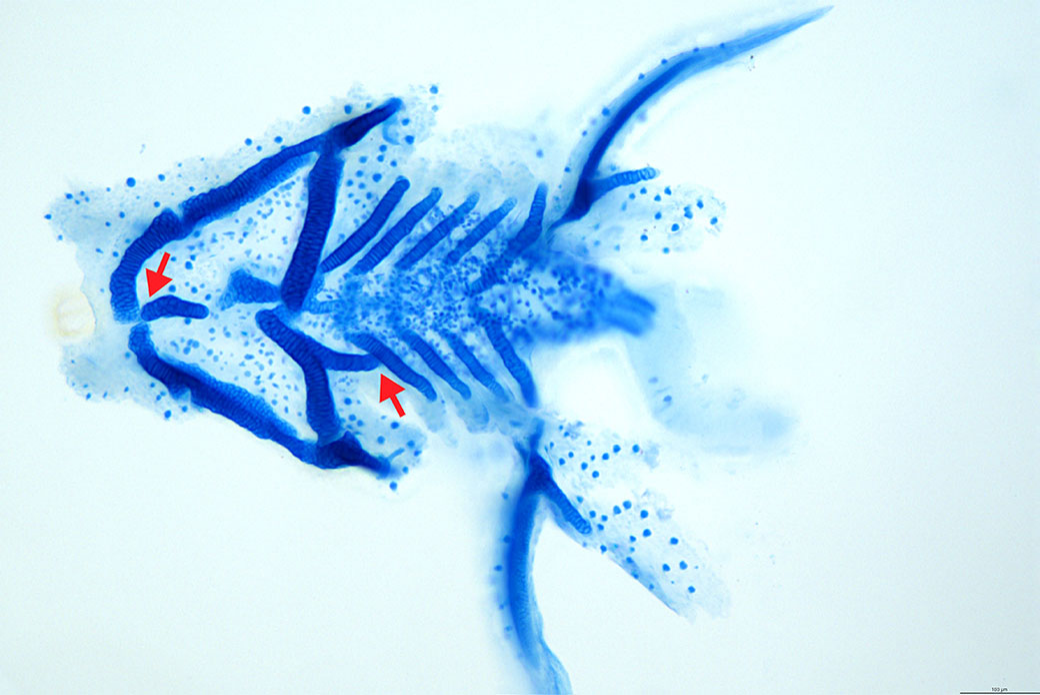
**Craniofacial cartilage dysmorphology observed in zebrafish larvae treated with elafibranor.** Hindered expansion and abnormal fusion of lower jaw structures (red arrows) are possible sequelae of elafibranor-induced epithelial constriction.

**What are the potential implications of these results for disease biology and the possible impact on patients?**

Our team is excited to have uncovered a novel potential role for IRF6 in promoting junctional protein localization. These findings provide new insights into the pathogenesis of skin barrier defects and epithelial anomalies present in syndromic orofacial clefts attributable to *IRF6*, as well as identify a potential drug target for mitigation of these phenotypes.

**Why did you choose DMM for your paper?**

DMM is a well-respected, open-access journal that offers high visibility for non-mammalian models of disease. Additionally, DMM has a thorough review process that provides constructive feedback, which I found especially helpful as an early-stage researcher.

**Given your current role, what challenges do you face and what changes could improve the professional lives of other scientists in this role?**

As a medical student researcher, I have worked in both clinical and basic science research settings and have observed persistent silos between the two. Successful translational research depends on close collaboration and shared expertise across these domains. One effective way to foster such collaboration is through conferences and seminar series that bring clinicians and scientists together, creating opportunities for mutual learning, relationship building and the development of joint research initiatives.

**What's next for you?**

Following the conclusion of my research fellowship in the Liao Laboratory, I will return to medical school at the University of Pennsylvania. I hope to follow in my grandmother's footsteps, becoming a plastic surgeon and physician-scientist, integrating groundbreaking translational research discoveries with innovative approaches to patient care.

**Tell us something interesting about yourself that wouldn't be on your CV**

I play the drums!
